# A Narrative Review of the Literature on Insufficient Sleep, Insomnia, and Health Correlates in American Indian/Alaska Native Populations

**DOI:** 10.1155/2019/4306463

**Published:** 2019-07-08

**Authors:** Anayansi Lombardero, Ciara D. Hansen, Andrew E. Richie, Duncan G. Campbell, Aaron W. Joyce

**Affiliations:** ^1^University of Alaska Anchorage, Anchorage, AK, USA; ^2^Waianae Coast Comprehensive Health Center, Waianae, HI, USA; ^3^University of Montana, Missoula, MT, USA; ^4^New Mexico VA Health Care System, Albuquerque, NM, USA

## Abstract

Insufficient sleep and insomnia promote chronic disease in the general population and may combine with social and economic factors to increase rates of chronic health conditions among AI/AN people. Given that insufficient sleep and insomnia can be addressed via behavioral interventions, it is critical to understand the prevalence and correlates of these disorders among AI/AN individuals in order to elucidate the mechanisms associated with health disparities and provide guidance for subsequent treatment research and practice. We reviewed the available literature on insufficient sleep and insomnia in the AI/AN population. PubMed, PsycINFO, Google Scholar, and ProQuest were searched between June 12^th^ and October 28^th^ of 2018. Prevalence of insufficient sleep ranged from 15% to 40%; insomnia prevalence ranged from 25% to 33%. Insufficient sleep was associated with unhealthy diet, low physical activity levels, higher BMI, worse self-reported health, increased risk for diabetes mellitus, cardiovascular disease, frequent mental distress, smoking, binge drinking, depression, and chronic pain. Insomnia was associated with depression, childhood abuse, PTSD, anxiety, alcohol use, low social support, and low trait-resilience levels. Research on evidence-based treatment and implementation practices targeting insufficient sleep and insomnia was lacking, and only one study described the development/validation of a measure of insufficient sleep among AI/AN people. There is a need for rigorous sleep research including testing and implementation of evidence-based treatment for insufficient sleep and insomnia in this population in an effort to help eliminate health disparities. We present recommendations for research and clinical practice based on the current review.

## 1. Introduction

Insufficient sleep and insomnia are associated with multiple medical and mental health problems, including increased risk for psychiatric disorders, suicide, and chronic health conditions such as obesity, diabetes, cardiovascular disease, and chronic pain [1–15]. In AI/AN populations, cardiovascular disease is among the leading causes of death [16], with experiences of social injustice and discrimination contributing to significant health disparities [17]. Many of the health conditions associated with insufficient sleep and insomnia are prevalent among AI/AN people [18]. This highlights a critical need to understand the possible role of sleep duration and quality caused by insomnia and insufficient sleep on the chronic conditions prevalent in AI/AN populations. It has recently been suggested that insufficient sleep and insomnia may help explain the disparities in cardiovascular disease among ethnic minority groups and that screening for and treating insomnia and insufficient sleep may aid in the prevention of cardiovascular disease [19].

*Insufficient sleep*, prevalent in 35% of the U.S. population [20], is defined as sleeping less than the 7-8 hours recommended by the American Academy of Sleep Medicine and the Sleep Research Society for optimal health [21]. *Insufficient sleep syndrome* (ISS) is considered a disorder in the International Classification of Disorders (ICD-11), under the classification of hypersomnolence disorders [22]. Walia and Mehra explain that ISS is characterized by sleep restriction that is self-induced (e.g., the person does not prioritize sleep) [23], in contrast to *insomnia disorder*, present in approximately 27% of US adults [24], which is defined by difficulty initiating or maintaining sleep with associated distress or impairment [25]. *Insomnia symptoms* refer to symptoms of insomnia, such as difficulty initiating or maintaining asleep that may not meet diagnostic criteria for insomnia disorder, with prevalence estimates ranging from 22% to 42% [26, 27]. These somewhat overlapping yet distinct problems with sleep quantity and quality represent a public health burden [28, 29].

Behavioral treatment of insomnia and insufficient sleep has potential to reduce the public health burden in a safe and cost-effective manner [30–32]. Cognitive Behavioral Therapy for Insomnia or CBT-I, for example, has proven to be an effective treatment for insomnia [33–36]. CBT-I addresses insomnia by use of stimulus control techniques (helping the patient associate the bed with sleep rather than wakefulness), sleep restriction strategies (initially reducing the amount of time spent in bed in order to consolidate sleep), and techniques that challenge thoughts that interfere with sleep (e.g., “If I do not get to sleep right now, I'll have a terrible day tomorrow”). CBT-I is as effective as pharmacological treatment in the short-term treatment of insomnia and more effective in the long-term treatment of insomnia [37], and it is now endorsed by the American College of Physicians as the first line of treatment for insomnia [38].

Regarding insufficient sleep, researchers have recommended that it be considered a behavioral risk factor [19], with potential to be addressed at both the individual and the population level via public advocacy and employer programs [39]. Although the research evidence for behavioral treatments for insufficient sleep is not as extensive as that for CBT-I, there is promise for sleep education programs. For example, some of these programs have resulted in increased sleep quality, sleep hygiene behaviors, increased sleep time on school nights, and reduced daytime sleepiness [32, 40]. Insufficient sleep can also be addressed via motivational interviewing (MI) [41], and MI has been found to be an effective tool for affecting health behavior change [42, 43].

Given that evidence-based treatments for insomnia and behavioral and educational strategies to address insufficient sleep have potential to mitigate health problems, it is important to assess whether an indication for these treatments exists among AI/AN individuals and the degree to which these treatments have been tested in this population. The aim of this paper is to review the literature on the prevalence, correlates, predictors, and treatment of insufficient sleep and insomnia among Al/AN people, with the goal of helping clarify the potential contribution of sleep in health disparities. Another aim of the present review is to provide recommendations for clinicians and researchers regarding considerations for sleep treatment and research of insufficient sleep in this population. Ultimately, systems-level approaches to insufficient sleep and insomnia have potential to lessen the rates of chronic disease and help eliminate health disparities among AI/AN populations.

## 2. Methods

**Figure 1:**
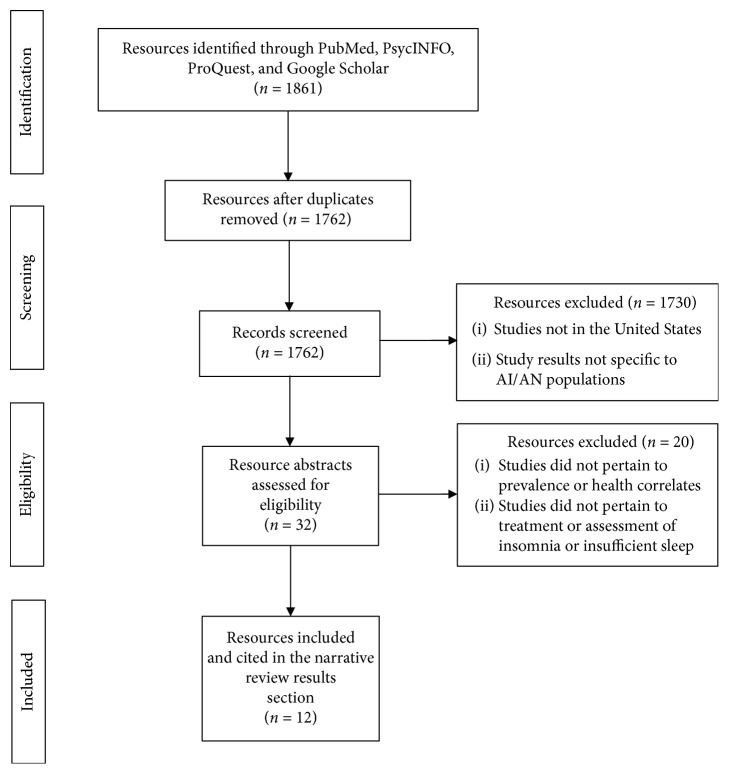
Resource identification and screening process.

The current study consisted of a narrative review, a synthesis of the literature, based on narrative review methodology described elsewhere [44, 45]. Our research team collaborated with a social sciences librarian to search and review the existing literature using PubMed, PsycINFO, ProQuest Journals, and Google Scholar for articles related to the prevalence and treatment of insufficient sleep in indigenous populations. Searches were conducted between June 12^th^ and October 28^th^ of 2018 with no specified date parameters. We included three publication types: peer-reviewed scholarly journals, government publications, and dissertations and theses in order to reduce possible effects of publication bias. The following search terms were used: Native American, American Indian, Alaska Native, Indigenous Populations, Insomnia, Sleep, and Sleep Disturbances (see Figure 1 for flow chart illustrating our search strategy).

We included articles that were written in English and conducted in the United States and that provided information on reported prevalence, and/or treatment therapeutics or assessment, of insufficient sleep, and insomnia, or symptoms of insomnia, in AI/AN populations. Sources that focused on indigenous populations outside of the United States, other sleep problems including parasomnias, circadian rhythm disorders, and breathing-related sleep disorders, or where AI/AN populations were included as part of their sample but did not describe data specific to each population were excluded. The rationale for the inclusion/exclusion criteria is that insufficient sleep and insomnia are sleep conditions that can be addressed via behavioral interventions and that do not require specialty equipment, such as continuous positive airway pressure technology or light therapy, as other sleep disorders might (e.g., sleep apnea and circadian rhythm disorders). In addition, we chose to focus on American Indian and Alaska Native populations rather than indigenous groups in other countries because our goal was to generate information that would be useful for systems-level approaches to research and intervention relevant to Indian Health Services and Tribal Health Organizations in the United States.

Our research team identified 1762 sources for review, after removing duplicates. The team (A.L., C.H., D.C., A.J., and A.R.) inspected the titles of each, selecting 32 that appeared to meet the inclusion criteria outlined in this section. Each investigator carefully read each of the 32 sources, collectively identifying 12 that provided relevant information on the reported prevalence or treatment therapeutics of insomnia and insufficient sleep in AI/AN populations. All team members read each of the included articles in their entirety and agreed that they should be included in the review. No additional articles were added after reviewing the references of selected papers.

## 3. Results

Our search yielded 12 articles that met criteria for inclusion. This small number of works indicated a dearth of knowledge regarding important aspects of insomnia among AI/AN populations, such as whether established measures of insomnia are culturally appropriate for use among AI/AN populations, the extent to which psychological/behavioral approaches for insomnia or insufficient sleep are effective in this population, and the prevalence of insomnia disorder in a representative AI/AN sample [47–59].  Table 1 presents a summary of sample descriptions, prevalence and correlates of insufficient sleep and insomnia, the measures used, and author recommendations for the 12 articles included in this review. The limited research literature focused on the following aspects of insufficient sleep and insomnia: (1) prevalence [20, 46–52], (2) demographic and cultural correlates [20, 46, 48–50, 53–55], (3) medical and mental health correlates and predictors [46–53, 55], and (4) theoretical mechanisms for the insufficient sleep-culture-chronic disease relationships [47–49].

There were no studies that examined rates of well-defined insomnia (either by use of a clinical interview or insomnia measure) in a representative sample of AI/AN individuals, although insomnia rates were reported for an AI/AN subpopulation of military personnel using a well-established insomnia measure, the Insomnia Severity Index (ISI) [50]. There was variability in the terminology used to describe insufficient sleep (e.g., short sleep duration and insufficient sleep) and insomnia (e.g., insomnia and insomnia symptoms). Insufficient sleep was defined as sleeping < 7 hours per day in most studies. One study used < 6 hours as a threshold for insufficient sleep [46], and none of the included studies used or mentioned ICD-11 criteria for ISS. Regarding assessment measure validity, a single study evaluated the validity of a newly developed sleep assessment measure [56]. The assessment measures used in the studies are listed in Table 1; only one of the included studies employed a measure that specifically assessed clinically significant insomnia (ISI) [50], and none used diagnostic interviews. Data from sleep diaries and from wearable sensor technology were reported in only one of the papers—an unpublished dissertation [54]. None of the studies focused on treatment for insomnia or insufficient sleep.

### 3.1. Prevalence of Insufficient Sleep and Insomnia

The majority of the literature appears to indicate a high prevalence of insufficient sleep among AI/AN populations. The nation-wide prevalence of healthy sleep duration was lower among AI/AN individuals compared to non-Hispanic White individuals (59.6% vs 66.8%), suggesting that the prevalence of unhealthy sleep duration (defined as < 7 hours per night) for AI/AN populations across the nation is approximately 40.4% [20]. Two other studies presented rates of insufficient sleep that were similar to or higher than those found in the general population: the first was a study conducted in a community sample that reported rates of insufficient sleep among their participants (*n* = 386) to be comparable to national averages, with 30% of the sample indicating sleeping < 7 hours per night and 15% reporting sleeping < 6 hours per night [46]. The second study used a much larger sample drawn from the Behavioral Risk Factor Surveillance Survey (*n* = 11,507) in which investigators found insufficient sleep (defined as believing one did not get enough sleep for 14 or more days during the past 30 days) to be higher among AI/AN populations compared to non-Hispanic White populations (34.2% vs 27.4%) [48].

In specific subsets of AI/AN populations, rates of insufficient sleep were even higher. More than 40% of a sample with prediabetes (*n* = 1,899), for example, reported sleeping ≤ 6 hours per night [47]. A dissertation project conducted among shiftwork nurses found a mean of 5 hours and 34 minutes of sleep in a 24-hour period for AI/AN participants and 6 hours and 10 minutes for White participants (prevalence of insufficient sleep was not reported in this sample) [54]. Of note, one study found obese participants to have higher prevalence of insufficient sleep among all races with the exception of AI/AN participants [48].

Regarding insomnia in special populations, rates do appear to be higher among AI/AN people compared to other groups. For example, among AI/AN army personnel, rates of insomnia as measured by the ISI were higher among AI/AN participants compared to White participants (33.7% vs 15.1%) [50]. For insomnia symptoms in AI/AN population subsets, a study among AI/AN people ages 55 and older found a 15% prevalence of participants with insomnia symptoms sleeping ≤ 5 hours per night [49]. AI/AN youth reported a 25% prevalence rate of insomnia (defined as experiencing sleep disturbance from “about once a week” to “everyday”). Lastly, among AI/AN individuals with PTSD, another one of the studies included in the present review found that 28.6% of Northern Plains AI Vietnam veterans with PTSD reported frequent trouble sleeping, an insomnia symptom [51].

In sum, the overall prevalence of insufficient sleep ranged from 15%, using conservative definitions (<6 hours of sleep per night) [46], to 40% when authors used more liberal definitions (<7 hours of sleep per night) [47]. No studies examined rates of well-defined insomnia (either by use of a clinical interview or insomnia measures) in a representative sample of AI/ANs, although among AI/AN military personnel and individuals with PTSD rates were between 28.6% and 33.7% [50, 51]. Variability in the ways authors determined insufficient sleep/sleep problems and inconsistent terminology make it difficult to determine exact rates in this population. In spite of this, the available literature suggests that insufficient sleep is a prevalent problem among AI/AN individuals [46] and that insomnia rates are higher among AI/AN military personnel compared to personnel of other ethnic groups [50].

### 3.2. Demographic, Physical, and Sociocultural Correlates of Insufficient Sleep and Insomnia

Demographic and sociocultural correlates of insufficient sleep among AI/ANs included older age [46, 49, 50], higher degrees of AI ancestry (but not identification with AI/AN culture) [46], and being unemployed or retired [47]. There were contradictory results for education levels, with one study finding insufficient sleep to be higher among those with a high school diploma vs those without one [46], and another study finding insomnia to be associated with lower education [49]. Among AI/AN active duty military personnel, insomnia was associated with recent stressful life events, higher rates of self-reported childhood abuse, lower social support, lower unit cohesion, lower trait resilience, longer military careers, more marriages, more military deployments, and more children [50].

The available literature identifies a plethora of sociocultural elements related to insufficient sleep and insomnia among AI/AN individuals. Although longitudinal and intervention studies demonstrating the effects of these factors on sleep are lacking, the studies in this review point to complex and possibly interplaying factors that are important to consider in prevention and treatment efforts. Further research examining sociocultural protective and risk factors are needed if researchers, clinicians, and systems are to incorporate these domains into prevention and treatment. In addition, longitudinal studies have potential to help in identifying and understanding relevant sociodemographic characteristics and their relationships with insufficient sleep and insomnia.

### 3.3. Medical and Mental Health Correlates and Predictors of Insufficient Sleep and Insomnia

Insufficient sleep was associated with a number of medical and psychological problems among AI/AN people. These include higher medical comorbidities [47], lower likelihood of reporting good or excellent health [47], diabetes [47], chronic pain and back pain [49], and cardiovascular disease [49]. Other correlates associated with health included higher body mass index [47], lower levels of physical activity [47, 48], and unhealthy diet consumption [47]. Mental health/substance use correlates of insufficient sleep included frequent mental distress [48], depression [49], anxiety [46], smoking [48, 49], and alcohol use disorders [53]. Insomnia was associated with greater PTSD symptomatology [50], depression [50, 52], anxiety [50], alcohol use [50], and lower trait resilience levels [50]. In a study conducted in an adult AI mental community sample (*n* = 386), mental health factors such as lifetime diagnoses of anxiety and affective disorders and substance use disorders were associated with insomnia symptoms but not with insufficient sleep (<6 hours) [46].

Insufficient sleep and insomnia also had differences in associations between depression and suicide: a study of 80 AI adolescents found less time in bed to be associated with increased odds of self-reported suicidality but not depression among AI adolescents [55]. A dissertation study using a sample of 232 students, ages 12–21, found insomnia to be a predictor of depression but not suicidal ideation or suicide attempts among AI/AN youth [52]. It is worth noting that the latter study operationalized insomnia with responses to a single item asking individuals whether they had trouble falling or staying asleep rather than with use of a validated insomnia measure. Given the high rates of suicide among AI/ANs, [57] it is exceedingly important to understand these associations. Further studies in this domain using well-validated measures are needed.

There were no longitudinal studies examining predictors of insufficient sleep or insomnia among AI/AN people. Alcohol use disorders [46], binge drinking [53], and affective disorders [46] predicted longer sleep latencies. Among AI/AN active duty military personnel, insomnia was predicted by greater PTSD, depression, fatigue, stressful life events, alcohol use, anxiety, extremity pain, history of head injury, childhood physical neglect, back pain, number of marriages, lower levels of social support/unit cohesion, and lower levels of tangible social support [50]. These sleep-health associations have implications for clinical interventions, including the need to address sleep in mental health and medical settings as a means of reducing the possible consequences of insufficient sleep and insomnia on mental and physical health. Another issue for the sleep clinician to consider is the degree to which physical or mental conditions interfere with sleep and the need to problem solve or refer patients to treatment, as appropriate. Clinicians should also keep open lines of communication with specialist providers for consultation if the conditions are still active.

### 3.4. Theoretical Mechanisms for the Insomnia and Insufficient Sleep Associations with Decreased Health

In order to help eliminate health disparities, it is important to advance our understanding of the mechanisms that drive the sleep-health relationship. The direction of the associations between insufficient sleep and decreased physical and mental health is not well understood [46], but both appear to be prevalent among AI/ANs [18, 48]. Although the relationship between insufficient sleep or short sleep duration, insomnia, and decreased health is a complex one [58], with insufficient sleep increasing the risk of health problems [14, 59] and health problems and behaviors contributing to insufficient sleep [53, 60], it is clear that chronic health conditions can develop or worsen once insufficient sleep patterns have been established [13, 14, 61]. Indeed, insufficient sleep is thought to play an important role in some of the health disparities found among American Indian/Alaska Native populations [47]. For example, insufficient sleep has demonstrated associations with increased diabetes risk among AI/ANs with prediabetes and with cardiovascular disease, which is among the leading causes of death among AI/AN people [62].

Proposed mechanisms for the insufficient sleep-diabetes and cardiovascular disease relationships among AI/ANs are thought to be similar to those in other ethnic/racial groups (i.e., short sleep duration affects hormonal and glycemic factors hindering regulation of hunger and contributing to obesity) [63, 64]. Chapman and colleagues suggest that sleep may be an underlying, unrecognized element in the increased risk for obesity, diabetes, and cardiovascular disease found among AI/ANs [48]. Sabanayagam and colleagues also propose that demographic and sociocultural characteristics should be understood as important factors affecting sleep-diabetes-cardiovascular disease relationships [49]. For example, the authors pose that multiple jobs and poor living environments affect sleep duration, making environmental stress the underlying cause of diabetes/heart disease via insufficient sleep [49]. It is crucial that these factors are researched and addressed in sleep studies, policy, and interventions in this population.

Regarding insufficient sleep, insomnia, and psychological distress, a bidirectional relationship has been posited by many researchers [65–67], including those in the current review [48]. Insomnia has been found to contribute to the development of PTSD [68] and depression [69]. In the present review, frequent mental distress was associated with insufficient sleep [48], suggesting that perhaps broader psychological distress contributes to insufficient sleep is the result of it or that it may serve as a potentiating ingredient. The majority of the literature in the present review focused on the insufficient sleep-health mechanism rather than insomnia, although some emphasized the need to examine the insomnia-psychiatric health association [50]. Indeed, insomnia symptoms were differentially linked to psychological distress as well as physical health. For example, in one study, sleep latency was predicted by alcohol use disorders and affective disorders, whereas night or early morning awakenings were associated with anxiety and affective disorders [46]. In addition, sleep-related breathing disorders (not examined in any of the current studies and beyond the scope of the current review) may also contribute to the health-insufficient sleep relationships [70, 71].

In examining these relationships, it can be important to distinguish each of the factors of sleep quality and how they directly affect physical and mental health. For the current study, we narrowed sleep difficulties to insufficient sleep and insomnia, as these can be readily treated via behavioral interventions. Additional author recommendations for research and practice based on the current review can be seen in Table 1. These recommendations include longitudinal research studies to determine the nature of the associations among sleep, mental and physical health, and sociocultural factors [46], the use of CBT-I and psychoeducation to address sleep problems among AI/ANs [48], and addressing sleep in AI/ANs with psychiatric [55] or chronic health problems [49] (see Table 1 for a detailed list of recommendations by study).

## 4. Discussion

The review of the literature on insufficient sleep and insomnia among AI/AN people yielded very few but instructive papers, highlighting that insufficient sleep and insomnia in this population represent important problems that need attention. Our review identified 12 studies that focused mostly on the examination of prevalence and correlates of insufficient sleep and insomnia in AI/AN populations. These articles yielded key information to consider in future research and clinical efforts for targeting insufficient sleep and insomnia among AI/AN people. Mainly, author recommendations for research and systems-level approaches to addressing these modifiable sleep disturbances include (1) longitudinal studies to clarify the associations between insufficient sleep, insomnia, and physical and psychological health problems; (2) assessment of insomnia and insufficient sleep, particularly in mental health settings; and (3) CBT-I and sleep education programs added to existing mental health and cardiovascular disease interventions (e.g., diabetes and weight loss programs); see Table 1 for more detailed recommendations.

Research was either lacking or very limited in several important areas of insufficient sleep and insomnia. Understudied issues included measure validation, cultural and socioeconomic predictors of insufficient sleep and insomnia, and treatment approaches for insomnia. For this reason, in addition to the author recommendations listed above, we would add the following recommendations for research: (1) development, testing, and use of well-validated measures of insomnia and insufficient sleep in AI/AN populations; (2) testing and implementation of CBT-I and educational programs for insufficient sleep in health settings and organizations serving AI/AN populations (e.g., Indian Health Services and Tribal Health Organizations), and (3) culturally tailoring interventions as needed to increase engagement in and completion of appropriate intervention programs.

In terms of addressing health disparities, first and foremost, we consider it important to highlight that many of the issues associated with health disparities among AI/AN people have important implications for policy and that valuable recommendations in this regard have been made elsewhere [18]. In addition, AI/AN people comprise many different cultures with both similar and distinct ideologies, environments, and histories [72]. Further, AI/AN individuals may have different levels of identification with their respective cultures and with the majority culture. Therefore, the recommendations we provide should be considered in light of these factors, and clinicians and researchers should be mindful of this and refrain from making generalizations or assumptions about their AI/AN patients or research participants. With that caution in mind, the recommendations we provide can be helpful to consider in insomnia and insufficient sleep research and clinical practice.

Given the modifiable nature of insufficient sleep and insomnia, one of the most important implications of our findings is the need for research and application of behavioral approaches to address these two sleep disturbances. We highlight behavioral interventions because of the increased risk for depression [73], suicide [74], accidents [75], and increased mortality [76] associated with prescription medication for insomnia. Important first steps toward testing and implementation of these approaches include conducting research examining the receptivity and fit of CBT-I and educational programs for insufficient sleep among AI/AN individuals and health settings serving AI/AN populations. Regarding CBT-I, studying openness to treatment as well as possible cultural adaptations is crucial in this population, as there may be important cultural factors not present in the majority culture in which CBT-I was tested that may affect outcomes in this population (e.g., some AI/AN languages do not have a word for insomnia) [77].

Another area of research in this domain includes the use of technology to deliver CBT-I, especially to those in rural areas or with limited access to behavioral health clinicians. Indeed, health technology has been recommended as a way to deliver CBT-I as a first line of treatment [78], and mobile and web-based interventions have already been tested and proven effective in non-native populations [79, 80]. In addition, systematic assessment of insomnia in primary care and mental health settings would help identify patients who could benefit from these interventions. Regarding insufficient sleep, it is imperative that it be addressed at both the population and the individual level. Although further research is needed in this domain, educational programs, motivational interviewing, and employer programs highlighting the crucial role of sleep on individuals' health have been proposed in the literature, as stated earlier. Some of these programs have demonstrated promise in increasing both sleep knowledge and quantity of sleep among adolescents.

In sum, our findings provide information that is valuable for systems-level approaches to addressing insufficient sleep and insomnia. In order to affect change, areas for further research identified in the current review include assessment of the causes of insomnia and insufficient sleep, as well as validation and/or creation of sleep assessment measures; cultural factors that may influence sleep and sleep treatment in this population; and treatment and prevention research. In particular, extension of the large body of research supporting the use of CBT-I in the general population to settings serving AI/AN populations would be a useful step toward promoting healthy sleep in a population that is potentially more vulnerable to insufficient sleep and insomnia and the negative physical and mental health sequelae.

## Figures and Tables

**Table 1:** Included articles.

Included articles (citation and class)	AI/AN sample description	Prevalence and correlates of insufficient sleep or insomnia	Sleep assessment measure	Author recommendations
(1) Ehlers et al. [46], peer-reviewed article	*n* = 386 AI/AN adult participants from 8 contiguous rural reservations	(i) Short sleep duration present in 15% for those sleeping <6 hours and 30% for those sleeping <7 was associated with age (>30), higher AI ancestry, and having a high school diploma. (ii) Lifetime diagnoses of substance use disorders, anxiety, and affective disorders were associated with higher PSQI cores. (iii) Sleep latency was predicted by alcohol use disorders and affective disorders; night/early morning awakenings were associated with anxiety and affective disorders. (iv) Anxiety and alcohol use disorders were associated with bad dreams.	(i) PSQI	(i) Longitudinal studies to determine the nature of the sleep-mental health disorder relationships. (ii) Assessment of bad dreams among AI/ANs as a culturally appropriate method of assessing for psychiatric and substance use disorders.

(2) Nuyujukian et al. [47], peer-reviewed article	*n* = 1,899 AI/AN adults with prediabetes from 36 health care programs in 18 states serving 80 tribes and 11 IHS areas	(i) Short sleep duration (present in more than 40% of the sample), but not long sleep duration, was associated with increased diabetes risk among AI/ANs with prediabetes. BMI and weight loss reduced these relationships so that they were no longer significant. (ii) Correlates of short sleep duration included the following: higher BMI, higher comorbidities, lower levels of physical activity, unhealthy diet, and worse self-reported health.	(i) Single item: “how many hours a night do you sleep on average?”	(i) The addition of sleep education to diabetes and weight loss problems. (ii) Further investigation into the reasons for the short sleep duration among AI/ANs.

(3) Chapman et al. [48], peer-reviewed article	*n* = 11,507 AI/AN adult participants from a national sample: BRFSS 2009–2010 data	(i) Prevalence of insufficient sleep was higher among AI/ANs compared to Whites (34.2% vs 27.4%). Frequent mental distress + demographics and obesity and lifestyle indicators reduced this relationship to nonsignificance. (ii) Insufficient sleep was associated with physical inactivity, frequent mental distress, smoking, and binge drinking.	(i) Single item: “during the past 30 days, for about how many days have you felt you did not get enough rest or sleep?”	(i) CBT-I and sleep education as interventions for addressing sleep to promote health and prevent disease among AI/ANs. (ii) Studies of health interventions addressing frequent mental distress among AI/ANs.

(4) Sabanayagam et al. [49], peer-reviewed article	*n* = 449 AI enrolled tribal members ages >55 from the US southeast region	(i) Short sleep duration prevalence was 15% and was associated with smoking, depression, chronic pain, and back pain; women reported difficulty falling asleep at higher rates than men. (ii) CVD was highest among those with short sleep duration and lowest among those sleeping seven hours after adjusting for confounds. (iii) Daytime sleepiness and difficulty falling asleep were also associated with CVD; with all three sleep variables taken into account, only daytime sleepiness was significantly associated with CVD.	Three items: (i) *Sleep duration*: “on average, how long do you sleep per night?” and “how many total hours do you sleep in an average 24-hour period?” (ii) *Daytime sleepiness*: “how often do you fall asleep during the day against your will?” (iii) Difficulty falling asleep: “how often do you have difficulty falling asleep or staying asleep?”	(i) Further studies in order to confirm findings. (ii) The addition of sleep interventions in CVD intervention programs among AI/ANs if current findings are confirmed by further studies.

(5) Ehlers et al. [53], peer-reviewed article	*n* = 219 AI participants, ages 18–30 from 8 contiguous reservations in the US	(i) Prevalence of short sleep duration was not reported. AI participants had higher overall PSQI scores, longer sleep latencies and longer sleep durations, and more problems with breathing and bad dreams compared to Mexican Americans. (ii) For the overall sample, binge drinking predicted longer sleep latencies, bad dreams, higher PSQI scores, and more problems with breathing after adjusting for demographics.	(i) PSQI	(i) Assessment of sleep quality, including difficulty falling asleep, sleep quality, and bad dreams in AI/AN young adults. (ii) Longitudinal studies examining associations between binge drinking and PSQI scores.

(6) Taylor et al. [50], peer-reviewed article	*n* = 74 AI/AN active duty service members	(i) Prevalence of insomnia was highest among AI/ANs (33.7% vs 15.1%–21.4% for other races/ethnicities) and were associated with older age, more military deployments, longer military careers, more marriages, and more children. (ii) Those with insomnia had higher rates of childhood abuse, more severe mental health symptoms, more recent stressful events, lower unit cohesion, and lower trait resilience levels. (iii) Predictors of insomnia (among all groups) after controlling for demographics included lower levels of social support, unit cohesion, childhood physical neglect, back pain, extremity pain, history of head injury, number of marriages, depression, PTSD severity, fatigue, anxiety, alcohol use, and stressful life events.	(i) ISI	None specific to AI/ANs; the following were made regarding military personnel: (i) Experimental, prospective, and intervention research in order to clarify the relationship the direction of the sleep-mental and physical health associations. (ii) Assessment and treatment of insomnia. (iii) Large-scale longitudinal studies to clarify relationships among sleep disorders, comorbidities, and correlates. (iv) Treatment studies that address the specific needs of military personnel.

(7) Arnold et al. [55], peer-reviewed article	*n* = 80 AI adolescents enrolled in the Lumbee tribe, ages 11–18	(i) Prevalence was not reported. Mean time in bed was 8.1 hours, with a range from 5–10 hours. Sleepiness was associated with depression but not suicidality while time in bed was not associated with depression but with decreased odds of suicidality; lower levels of connection to Lumbee culture and nonheterosexual orientation were associated with depression. (ii) Less time in bed and depression predicted suicidality; depression was associated with sleepiness, suicidality, and self-esteem.	(i) ESS (modified) (ii) *Two items measuring average time in bed:* “(1) what time do you usually go to bed if you have school the next morning?” “(2) what time do you usually wake up on school days?”	(i) Comprehensive assessment of sleep problems among AI youth with depression/suicide risk. (ii) Treatment of sleep problems in order to improve AI/AN mental health. (iii) Development of culturally sensitive interventions for depression and suicidality.

(8) Shore et al. [51], peer-reviewed article	*n* = 305 adult participants from the Northern Plains (PRA)	(i) Difficulty sleeping was reported by 28% of those with PTSD vs 13.4% for those with no trauma and 21% for those with at least one trauma. (ii) Those with combat-related PTSD indicated higher rates of difficulty sleeping (45.4% vs 25.4%) compared to the overall sample. (iii) No health correlates in addition to PTSD were reported.	(i) Five items from the Mississippi Scale for Combat-Related PTSD for difficulty sleeping and nightmares: trouble sleeping, nightmares, awakening due to nightmares, daydreams, and fear of going to sleep at night.	(i) Clinician awareness regarding high rate of nightmares and sleep disturbance among AI veterans. (ii) Clinician awareness of the importance of dreams for AIs as well as education regarding their patient's specific tribes and their beliefs about dreams. (iii) Assessment of every patient's cultural background including cultural identification, beliefs, and practices. (iv) Further research to improve the provision of culturally appropriate care by investigating the meaning, context, and frequency of nightmares among AI/ANs with trauma and PTSD. (v) Consideration of referrals to traditional healers or cultural consultants for those with strong AI/AN identification.

(9) Cook and Burd [56], peer-reviewed article	*n* = 115 AI/AN children, ages 7 to 20, from two reservation schools	(i) Prevalence of insomnia symptoms or insufficient sleep was not reported. Six factors emerged in factor analysis: (1) unusual or sensational behaviors (reluctance to accept the conventions of sleep),(2) insecure, fearful behaviors, (3) Daytime napping and fatigue, (4) waking up difficulties, (5) Physical discomfort and pain, and(6) difficulties falling asleep. (ii) Health correlates were not reported.	(i) PSDQ (scale items were taken from the SHQ, developed by one of the authors).	(i) Further research and refinement of the scale in an effort to improve diagnosis and treatment of sleep disturbance. (ii) The measure was found to be appropriate for use with AN/AI youth seen at the authors' clinic, but the investigators highlighted a need for refinement of their measure to improve diagnosis and treatment.

(10) Farrell [52], dissertation	*n* = 232 students, ages 12–21 from a national sample who self-identified as AI/AN	(i) 25% of participants were identified as having insomnia. (ii) “Insomnia” predicted depression but not suicidal ideation or suicide attempts in this sample.	(i) Single item from the General Health Questionnaire: “trouble falling asleep or staying asleep” during the past 12 months.	(i) Further research examining sleep and suicidality in AI/AN groups by region and in groups or communities with high suicide rates. (ii) Use of these research findings in prevention, assessment, and treatment of suicide among AI/AN adolescents.

(11) Liu et al. [20], government document	*n* = ∼11,500 AI/AN adult participants from a national sample: BRFSS 2014 data	(i) AI/AN participants had lower prevalence of healthy sleep duration compared to Whites (59.6% vs 66.8%). (ii) Health correlates were not reported.	(i) Single item: “on average, how many hours of sleep do you get in a 24-hour period?”	(i) Provision of healthy sleep through health education and behavior change. (ii) Address insomnia symptoms with improved sleep habits or psychological or behavioral therapies. (iii) Regarding sleep aids (either over the counter or prescription), the authors state that there are no recommendations by professional sleep organizations regarding efficacy or safety.

(12) Hobbs [54], dissertation	*n* = 58 AI/AN nurses, ages 25 to 61	(i) Prevalence of insufficient sleep was not reported. Sleep length (calculated by Actiware™ data using a subsample) was short for the overall sample (5 hours and 34 minutes for AI/AN and 6 hours and 10 minutes for White nurses); this difference was nonsignificant. (ii) Sleep efficiency ratio was lower for AI/AN nurses in the subsample; they had more frequent wake after sleep onset, reported more difficulty falling asleep, and took fewer and shorter naps compared to White nurses.	(i) ESS (ii) Actiwatch™ (iii) Activity and sleep diaries (iv) Eight self-report items assessing sleep habits: satisfaction with sleep, sleep quality, restfulness after sleep, waking up earlier than intended, difficulty falling asleep, and use of sleeping pills, over-the-counter sleep aids, or alcohol as a sleep aid. (v) Early/Late Preferences Scale	(i) Hospital administrators might want to consider the effects of short sleep duration on mistakes by nurses working nightshifts. (ii) Further studies examining situational sleepiness with larger, more diverse samples. (iii) Further studies examining the effects of tobacco on sleep among AI/AN nurses.

AI/AN = American Indian/Alaska Native; CVD = cardiovascular disease; PSQI = Pittsburgh Sleep Quality Index; ESS = Epworth Sleepiness Scale; ISI = Insomnia Severity Index; PSDQ = Pediatric Sleep Disturbance Questionnaire; SHQ = Sleep Habits Questionnaire; BRFSS = Behavioral Risk Factor Surveillance Survey.

## References

[1] Sivertsen B., Krokstad S., Overland S., Mykletun A. (2009). The epidemiology of insomnia: associations with physical and mental health: the HUNT-2 study. *Journal of Psychosomatic Research*.

[2] Biddle D. J., Kelly P. J., Hermens D. F., Glozier N. (2018). The association of insomnia with future mental illness: is it just residual symptoms?. *Sleep Health*.

[3] Tang W., Lu Y., Xu J. (2018). Post-traumatic stress disorder, anxiety and depression symptoms among adolescent earthquake victims: comorbidity and associated sleep-disturbing factors. *Social Psychiatry and Psychiatric Epidemiology*.

[4] Smith M. T., Haythornthwaite J. A. (2004). How do sleep disturbance and chronic pain inter-relate? Insights from the longitudinal and cognitive-behavioral clinical trials literature. *Sleep Medicine Reviews*.

[5] Roberts M. B., Drummond P. D. (2016). Sleep problems are associated with chronic pain over and above mutual associations with depression and catastrophizing. *Clinical Journal of Pain*.

[6] McCall W. V., Black C. G. (2013). The link between suicide and insomnia: theoretical mechanisms. *Current Psychiatry Reports*.

[7] Park W.-S., Yang K. I., Kim H. (2019). Insufficient sleep and suicidal ideation: a survey of 12,046 female adolescents. *Sleep Medicine*.

[8] Grandner M. A. (2017). Sleep and obesity risk in adults: possible mechanisms; contextual factors; and implications for research, intervention, and policy. *Sleep Health*.

[9] Cai G.-H., Theorell-Haglöw J., Janson C. (2018). Insomnia symptoms and sleep duration and their combined effects in relation to associations with obesity and central obesity. *Sleep Medicine*.

[10] Cappuccio F. P., D’Elia L., Strazzullo P., Miller M. A. (2010). Quantity and quality of sleep and incidence of Type 2 Diabetes: a systematic review and meta-analysis. *Diabetes Care*.

[11] Mallon L., Broman J.-E., Hetta J. (2005). High incidence of diabetes in men with sleep complaints or short sleep duration: a 12-year follow-up study of a middle-aged population. *Diabetes Care*.

[12] Zhang Y., Lin Y., Zhang J., Liu X., Wang T., Gao Z. (2019). Association between insomnia and type 2 diabetes mellitus in Han Chinese individuals in Shandong Province, China. *Sleep and Breathing*.

[13] Sofi F., Cesari F., Casini A., Macchi C., Abbate R., Gensini G. F. (2012). Insomnia and risk of cardiovascular disease: a meta-analysis. *European Journal of Preventive Cardiology*.

[14] Bertisch S. M., Pollock B. D., Mittleman M. A. (2018). Insomnia with objective short sleep duration and risk of incident cardiovascular disease and all-cause mortality: sleep Heart Health Study. *Sleep*.

[15] Vishnu A., Shankar A., Kalidindi S. (2011). Examination of the association between insufficient sleep and cardiovascular disease and diabetes by race/ethnicity. *International Journal of Endocrinology*.

[16] Heron M. (2018). Deaths: leading casues for 2016. *N. V. S. Report*.

[17] Ramona B., Schultz K., Fernandez A. R., Walters K. L., Duran B., Evans-Campbell T. (2018). From ambivalence to revitalization: negotiating cardiovascular health behaviors related to environmental and historical trauma in a Northwestern American Indian community. *American Indian and Alaska Native Mental Health Research*.

[18] Payne H. E., Steele M., Bingham J. L., Sloan C. D. (2018). Identifying and reducing disparities in mental health outcomes among American Indians and alaskan natives using public health, mental healthcare and legal perspectives. *Administration and Policy in Mental Health and Mental Health Services Research*.

[19] Grandner M. A., Alfonso-Miller P., Fernandez-Mendoza J., Shetty S., Shenoy S., Combs D. (2016). Sleep: important considerations for the prevention of cardiovascular disease. *Current Opinion in Cardiology*.

[20] Liu Y., Wheaton A. G., Chapman D. P., Cunningham T. J., Lu H., Croft J. B. (2016). Prevalence of healhty sleep duration among adults-United States, 2014. *Prevention USDHH CDC*.

[21] Watson N. F., Watson N. F., Badr M. S. (2015). Joint consensus statement of the American Academy of sleep medicine and sleep research society on the recommended amount of sleep for a healthy adult: methodology and discussion. *Sleep*.

[22] World Health Organization (2018). *ICD-11 for Mortality and Morbidity Statistics (ICD-11 MMS)*.

[23] Walia H. K., Mehra R. (2016). Overview of common sleep disorders and intersection with dermatologic conditions. *International Journal of Molecular Sciences*.

[24] Olfson M., Wall M., Liu S.-M., Morin C. M., Blanco C. (2018). Insomnia and impaired quality of life in the United States. *Journal of Clinical Psychiatry*.

[25] American Psychiatric Association (2013). *Diagnostic and Statistical Manual of Mental Disorders*.

[26] Blanck M., Zhang J., Lamers F., Taylor A. D., Hickie I. B., Merikangas K. R. (2015). Health correlates of insomnia symptoms and comorbid mental disorders in a nationally representative sample of US adolescents. *Sleep*.

[27] Zheng W., Luo X.-N., Li H.-Y. (2018). Prevalence of insomnia symptoms and their associated factors in patients treated in outpatient clinics of four general hospitals in Guangzhow, China. *BMC Psychiatry*.

[28] Morin C. M., Jarrin D. C. (2013). Epidemiology of insomnia. *Sleep Medicine Clinics*.

[29] Grandner M. A., Pack A. I. (2011). Sleep disorders, public health, and public safety. *JAMA*.

[30] De Bruin E. J., van Steensel F. J. A., Meijer A. M. (2016). Cost-Effectiveness of group and Internet cognitive behavioral therapy for insomnia in adolescents: results from a randomized controlled trial. *Sleep*.

[31] MC S., Bramoweth A. D., Williams J., Roth A., Mosti C. (2014). Impact of brief cognitive behavioral treatment for insomnia on health care utilization and costs. *Journal of Clinical Sleep Medicine*.

[32] Tamura N., Tanaka H. (2016). Effects of a sleep education program with self-help treatment on sleeping patterns and daytime sleepiness in Japanese adolescents: a cluster randomized trial. *Chronobiology International*.

[33] Koffel E. A., Koffel J. B., Gehrman P. R. (2015). A meta-analysis of group cognitive behavioral therapy for insomnia. *Sleep Medicine Reviews*.

[34] Okajima I., Komada Y., Inoue Y. (2010). A meta-analysis on the treatment effectiveness of cognitive behavioral therapy for primary insomnia. *Sleep and Biological Rhythms*.

[35] Trauer J. M., Qian M. Y., Doyle J. S., Rajaratnam S. M. W., Cunnington D. (2015). Cognitive behavioral therapy for chronic insomnia. *Annals of Internal Medicine*.

[36] Johnson J. A., Rash J. A., Campbell T. S. (2016). A systematic review and meta-analysis of randomized controlled trials of cognitive behavior therapy for insomnia (CBT-I) in cancer survivors. *Sleep Medicine Reviews*.

[37] Riemann D., Perlis M. L. (2009). The treatments of chronic insomnia: a review of benzodiazepine receptor agonists and psychological and behavioral therapies. *Sleep Medicine Reviews*.

[38] National Institures of health state-of-the-science conference statement on manifestations and management of chronic insomnia in adults. NIH Consens State Sci Statements, 2005 17308547

[39] Chattu V. K., Sakhamuri S. M., Kumar R., Spence D. W., BaHammam A. S., Pandi-Perumal S. R. (2018). Insufficient sleep syndrome: is it time to classify it as a major noncommunicable disease?. *Sleep Science*.

[40] Brown F. C., Buboltz W. C., Soper B. (2006). Development and evaluation of the sleep treatment and education program for students (STEPS). *Journal of American College Health*.

[41] Gold M. A., Dahl R. E., Perlis M., Aloia M., Kuhn B. (2011). Using motivational interviewing to facilitate healthier sleep-related behaviors in adolescents. *Behavioral Treatments for Sleep Disorders: Practical Resources for the Mental Health Professional*.

[42] Gayes L. A., Steele R. G. (2014). A meta-analysis of motivational interviewing interventions for pediatric health behavior change. *Journal of Consulting and Clinical Psychology*.

[43] O’Halloran P. D., Blackstock F., Shields N. (2014). Motivational interviewing to increased physical activity in people with chronic health conditions: a systematic review and meta-analysis. *Clinical Rehabilitation*.

[44] Green B. N., Johnson C. D., Adams A. (2006). Writing narrative literature reviews for peer-reviewed journals: secrets of the trade. *Journal of Chiropractic Medicine*.

[45] Ferrari R. (2015). Writing narrative style literature reviews. *Medical Writing*.

[46] Ehlers C. L., Wills D. N., Lau P., Gilder D. A. (2017). Sleep quality in an adult American Indian community sample. *Journal of Clinical Sleep Medicine*.

[47] Nuyujukian D. S., Beals J., Huang H. (2016). Sleep duration and diabetes risk in American Indian and Alaska native participants of a lifestyle intervention project. *Sleep*.

[48] Chapman D. P., Croft J. B., Liu Y., Perry G. S., Presley-Cantrell L. R., Ford E. S. (2013). Excess frequent insufficient sleep in American Indians/Alaska natives. *Journal of Environmental and Public Health*.

[49] Sabanayagam C., Shankar A., Buchwald D., Goins R. T. (2011). Insomnia symptoms and cardiovascular disease among older American Indians: the Native Elder Care Study. *Journal of Environmental and Public Health*.

[50] Taylor D. J., Pruiksma K. E., Hale W. J. (2016). Prevalence, correlates, and predictors of insomnia in the US Army prior to deployment. *Sleep*.

[51] Shore J. H., Orton H., Manson S. M. (2009). Trauma-related nightmares among American Indian Veterans: views from the dream catcher. *American Indian and Alaska Native Mental Health Research*.

[52] Farrell E. I. (2013). *Sleep Disturbance as an Independent Predictor of Suicidality in American Indian/Alaska Native Adolescents, ProQuest: Psychology*.

[53] Ehlers C. L., Wills D., Gilder D. A. (2018). A history of binge drinking during adolescence is associated with poorer sleep quality in young adult Mexican Americans and American Indians. *Psychopharmacology*.

[54] Hobbs B. B. (2004). *Individual Differences and Sleep Disturbances in American Indian/Alaska Native and White Non-Hispanic Nurse Shiftworkers. ProQuest: Nursing*.

[55] Arnold E. M., McCall V. W., Anderson A., Bryant A., Bell R. (2013). Sleep problems, suicidality and depression among American Indian youth. *Journal of Sleep Disorders: Treatment & Care*.

[56] Cook J., Burd L. (1990). Preliminary report on construction and validation of a pediatric sleep disturbance questionnaire. *Perceptual and Motor Skills*.

[57] Herne M. A., Bartholomew M. L., Weahkee R. L. (2014). Suicide mortality among American Indians and Alaska natives, 1999–2009. *American Journal of Public Health*.

[58] Javaheri S., Redline S. (2017). Insomnia and risk of cardiovascular disease. *Chest*.

[59] He Q., Zhang P., Li G., Dai H., Shi J. (2014). The associations between insomnia symptoms and risk of cardio-cerebral vascular events: a meta analysis of prospective studies. *European Journal of Preventive Cardiology*.

[60] Koyanagi A., Garin A., Olaya B. (2014). Chronic conditions and sleep problems among adults aged 50 years or over in nine countries: a multi-country study. *PLoS One*.

[61] Blank M., Zhang J., Lamers F., Taylor A. D., Hickie I. B., Merikangas K. R. (2015). Health correlates of insomnia symptoms and comorbid mental disorders in a nationally representative sample of US adolescents. *Sleep*.

[62] Espey D. K., Jim M. A., Cobb N. (2014). Leading causes of death and all-cause mortality in American Indians and Alaska Natives. *American Journal of Public Health*.

[63] Ip M., Mokhlesi B. (2007). Sleep and glucose intolerance/diabetes mellitus. *Sleep Medicine Clinics*.

[64] Spiegel K., Tasali E., Penev P., Cauter E. V. (2004). Brief communication: sleep curtailment in healthy young men is associated with decreased leptin levels, elevated Ghrelin levels, and increased hunger and appetite. *Annals of Internal Medicine*.

[65] Krystal A. D. (2012). Psychiatric disorders and sleep. *Neurologic Clinics*.

[66] Kahn M., Sheppes G., Sadeh A. (2013). Sleep and emotions: bidirectional links and underlying mechanisms. *International Journal of Psychophysiology*.

[67] Cousins J. C., Whalen D. J., Dahl R. E. (2011). The bidirectional association between daytime affect and nighttime sleep in youth with anxiety and depression. *Journal of Pediatric Psychology*.

[68] Wright K. M., Britt T. W., Bliese P. D., Adler A. B., Picchioni D., Moore D. (2011). Insomnia as predictor versus outcome of PTSD and depression among Iraq combat veterans. *Journal of Clinical Psychology*.

[69] Taylor D. J. (2008). Insomnia and depression. *Sleep*.

[70] Sharafkhaneh A., Giray N., Richardson P., Young T., Hirshkowitz M. (2005). Association of psychiatric disorders and sleep apnea in a large cohort. *Sleep*.

[71] Punjabi N. M., Caffo B. S., Goodwin J. L. (2009). Sleep-disordered breathing and mortality: a prospective cohort study. *PLoS Medicine*.

[72] Fleming C. M., Orlandi M. A., Weston R., Epstein L. G. (1992). American Indian and Alaska Natives: changing societies past and present. *Cultural Competence for Evaluators: A Guide for Alcohol and Other Drug Abuse Prevention Practitioners Working with Ethnic/Racial Communities, OSAP Cultural Competence Series, 1*.

[73] Kripke D. F. (2007). Greater incidence of depression with hypnotic use than with placebo. *BMC Psychiatry*.

[74] Voaklander D. C., Rowe B. H., Dryden D. M., Pahal J., Saar P., Kelly K. D. (2008). Medical illness, medication use and suicide in seniors: a population-based case-control study. *Journal of Epidemiology & Community Health*.

[75] Wang P. S., Bohn R. L., Glynn R. J., Mogun H., Avorn J. (2001). Zolpidem use and hip fractures in older people. *Journal of the American Geriatrics Society*.

[76] Kripke D. F., Klauber M. R., Wingard D. L., Fell R. L., Assmus J. D., Garfinkel L. (1998). Mortality hazard associated with prescription hypnotics. *Biological Psychiatry*.

[77] Berardinelli C. G. (1997). *The Health Care Experiences and Health-Seeking Patterns of Native Americans in an Urban Environment*.

[78] Espie C. A. (2009). Stepped care: a health technology solution for delivering cognitive behavioral therapy as a first line insomnia treatment. *Sleep*.

[79] Ye Y.-y., Chen N.-k., Chen J. (2016). Internet-based cognitive-behavioural therapy for insomnia (ICBT-i): a meta analysis of randomised controlled trials. *BMJ Open*.

[80] Shin J. C., Kim J., Grigsby-Toussaint D. (2017). Mobile phone interventions for sleep disorders and sleep quality: systematic review. *JMIR mHealth and uHealth*.

